# Photo‐Curable Stretchable High‐k Polymer/TiO_2_ Nanosheet Hybrid Dielectrics for Field‐Effect Transistors

**DOI:** 10.1002/smsc.202400197

**Published:** 2024-09-10

**Authors:** Qun‐Gao Chen, Xingke Cai, Chu‐Chen Chueh, Wen‐Ya Lee

**Affiliations:** ^1^ Department of Chemical Engineering and Biotechnology National Taipei University of Technology Taipei 106 Taiwan; ^2^ Institute for Advanced Study Shenzhen University Shenzhen 518060 P. R. China; ^3^ Department of Chemical Engineering National Taiwan University Taipei 10617 Taiwan

**Keywords:** dielectrics, nanocomposites, stretchable field‐effect transistor, TiO_2_ nanosheets

## Abstract

Elastomeric polymer materials are of interest due to their stretchability, low‐temperature processing, and scalability. In addition, the incorporation of 2D materials can further enhance the dielectric properties and capacitance of elastic polymer materials, thereby reducing the driving voltage and energy consumption. In this study, titanium dioxide (TiO_2_) nanosheets are cross‐linked with nitrile butadiene rubber using thiol‐ene click chemistry, which leads to the preparation of nanocomposite dielectric films with stretchability and high dielectric constant. Furthermore, by controlling the doping amount of the nanosheets, it is observed that the capacitance of the nanocomposite films increases from 25.61 to 684.67 nF cm^−2^, and the dielectric constant increases from 14.96 to 161.98. Finally, the stretchable nanocomposite films exhibit good insulating properties even at 50% strain. In this study, insight is provided into the potential of in situ cross‐linking between elastic polymer materials and 2D materials to produce high‐k dielectric materials with both stretchability and high insulating properties.

## Graphical abstract

The stretchable capacitor is composed of elastomer and TiO_2_ nanosheets through cross‐linking reaction produces dipole polarization under the influence of bias, and the doping of TiO_2_ nanosheets aggravates the polarization characteristics and significantly enhances the insulator properties such as the capacitance and the dielectric constant of the stretchable nanocomposite dielectric film.

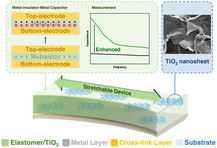

## 1 Introduction

The use of polymer materials in stretchable field‐effect transistors (FETs) has become a clear trend in recent years due to their excellent mechanical flexibility, low‐temperature processability, and suitability for large‐scale manufacturing in wearable electronics. However, conventional silicon‐based components are mechanically brittle and unsuitable for stretchable circuits. Consequently, there is a proliferation of techniques to develop stretchable electronic components, including rigid‐island architectures,^[^ ^1^, ^2^ ^]^ buckled surfaces,^[^ ^3^, ^4^ ^]^ and intrinsically stretchable materials. Among these approaches, the development of intrinsically stretchable materials is regarded as the most promising. Unlike rigid‐island devices with reduced device density and buckled‐surface devices with rough and wrinkled surfaces, intrinsically stretchable materials have the potential to be suitable for large‐scale high‐density devices.

Nowadays, some research groups utilize photo‐cross‐linking techniques to develop finely patterned electronic devices via rigid islands.^[^ ^5^, ^6^ ^]^ Even if the optically selective cross‐linking techniques for patterning are currently being developed, rigid islands remain a challenge to develop sufficiently patterned devices and high‐density electronics. However, several research groups have reported conjugated polymer chain modification processes and cross‐linked networks to develop intrinsically stretchable semiconductors.^[^ ^7^, ^8^, ^9^, ^10^, ^11^ ^]^ Moreover, to meet the demands of wearable electronic devices, we require not only intrinsically stretchable semiconductor materials but also high dielectric constant insulating materials that can withstand deformation. In addition to excellent insulating properties, an ideal dielectric must have a high dielectric constant so that energy consumption due to excessive leakage currents can be greatly reduced, which is a major challenge for device miniaturization. There is a growing number of reports on the utilization of high‐permittivity stretchable polymer dielectrics.^[^ ^12^, ^13^, ^14^ ^]^ A stretchable transistor based on carbon nanotubes (CNTs) and thermoplastic polyurethane (TPU) dielectric was reported by Chortos et al.^[^ ^15^ ^]^ CNTs were sprayed on the TPU dielectric for FET fabrication. Quasi‐static capacitance measurements with strain were performed in the CNT/TPU/CNT capacitor. The capacitance varies little with strain, and the capacitance of the CNT/TPU/CNT capacitor can be stabilized at about 30–36 nF cm^−2^. Previously, we reported a stretchable polymer capacitor based on a blend of poly(vinylidene fluoride‐hexafluoropropylene) and poly(4‐vinylphenol) as the dielectric material with a capacitance as high as 34.6 nF cm^−2^. The dielectric constant showed a variation from 5 to 10 with the ratio of the blends. By realizing high dielectric constants, we enabled the polymer FET to operate at smaller threshold voltages (*V*_TH_) and at voltages below 5 V. In addition, the average mobility (*μ* ^avg^) of the device during stretching is ≈0.1 cm^2^ V^−1^ s^−1^.^[^ ^16^ ^]^ Recently, the Bao's research group reported soft e‐skins based on a tri‐layer high‐permittivity elastomeric dielectric consisting of poly(acrylonitrile*‐*co‐butadiene) rubber (NBR), poly(styrene‐b‐ethylene‐co‐butylene‐b‐styrene), and octadecyltrimethoxysilane. The capacitance of the dielectric was 51 nF cm^−2^ and the dielectric constant was about 28. The organic field‐effect transistor (OFET) based on this tri‐layer dielectric showed an average mobility of 0.7 cm^2^ V^−1^ s^−1^ at an actuation voltage of −3 V.^[^ ^17^ ^]^

Although many reports have emphasized the excellent stretchability of polymer dielectrics, their relative dielectric constant values (6–20) are still much lower compared to inorganic materials (>100). By blending inorganic materials with polymers and improving film stability through cross‐linking reactions, nanocomposite dielectric films with both flexibility and high dielectric constants can be generated.^[^ ^18^ ^]^ Notably, titanium dioxide (TiO_2_) is of interest due to its ability to participate in redox reactions and its high‐permittivity properties (*ε* = 100–120).^[^ ^19^, ^20^, ^21^ ^]^ TiO_2_ has been widely used in photocatalysis,^[^ ^22^, ^23^, ^24^, ^25^ ^]^ electronic devices,^[^ ^26^, ^27^, ^28^, ^29^, ^30^, ^31^ ^]^ dielectrics,^[^ ^32^, ^33^, ^34^ ^]^ and energy‐storage devices.^[^ ^35^, ^36^, ^37^, ^38^ ^]^ Recently, in dielectric studies based on 2D materials, Tian et al. combined Ti_0.87_O_2_ monolayers with polypropylene (PP) using a freeze‐drying and hot‐pressing process to further improve the dielectric properties.^[^ ^33^ ^]^ Jang et al. reported a method of drop‐casting poly(vinyl alcohol) (PVA) on TiO_2_ nanosheets to generate TiO_2_ nanosheets–PVA nanocomposite dielectrics, which improved the insulation characteristics.^[^ ^18^ ^]^ In addition, TiO_2_ has been found to react with thiol‐alkenes, where photoexcited holes generated in the TiO_2_ conduction band react with the thiol group (—SH) to form thiyl radicals. Subsequently, these radicals undergo radical cross‐linking reactions with the alkene.^[^ ^39^, ^40^, ^41^, ^42^ ^]^ Although TiO_2_‐based nanocomposite films have been reported, these inorganic nanocomposites have limitations in terms of mechanical properties of the composite films.

**Figure 1:**
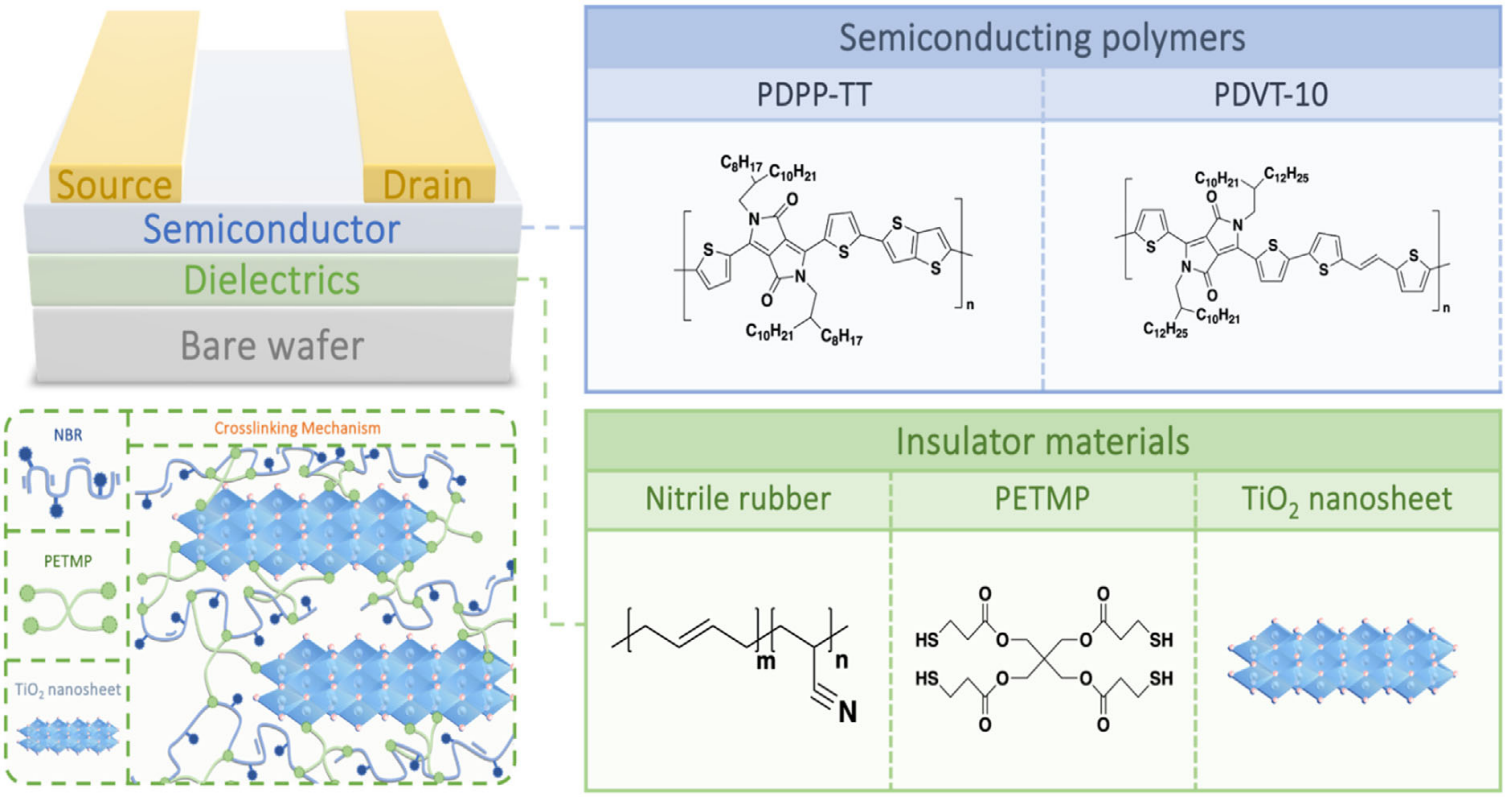
Schematic diagram of the cross‐linking mechanism and chemical structures of insulator materials and conjugated polymers.

To improve the mechanical robustness and insulating properties of composite films for stretchable electronic applications, we incorporated TiO_2_ nanosheets into nitrile rubber (NBR) via a photoinduced thiol‐ene click chemistry to form a cross‐linked structure. The cross‐linked embedded 2D polymer composites enhance the insulating characteristics of the nanocomposite dielectric while improving the stretchability. This approach aims to develop nanocomposite dielectric films suitable for wearable electronic devices. In this work, we prepared nanocomposite dielectric films consisting of TiO_2_ nanosheets and NBR using free radical cross‐linking reaction (**Figure** **1**). We investigated the insulating properties and surface morphology of the films and analyzed the crystalline structure of the 2D materials in the cross‐linked polymer system using grazing incidence X‐ray diffraction (GIXD) analysis. Finally, we demonstrated the electrical properties of stretchable nanocomposite dielectric films when applied to polymer FETs. By depositing PDPP‐TT (Poly{2,2′‐[(2,5‐bis(2‐octyldodecyl)‐3,6‐dioxo‐2,3,5,6‐tetrahydropyrrolo[3,4‐c]pyrrole‐1,4‐diyl)]dithiophene‐5,5′‐diyl‐alt‐thieno[3,2‐b]thiophen‐2,5‐diyl})‐ and PDVT‐10 (Poly{3,6‐dithiophen‐2‐yl‐2,5‐di(2‐decyltetradecyl)‐pyrrolo[3,4‐c]pyrrole‐1,4‐dione‐alt‐thienylenevinylene‐2,5‐yl}‐conjugated polymers on nanocomposite dielectric films doped with TiO_2_ nanosheets at a concentration of 30 vol% (Figure 1), we obtained transconductance of 0.202 and 0.808 μS along with a current on/off ratio of 10^5^, respectively. This study provides valuable insights into stretchable organic/inorganic composite dielectric films with high permittivity.

## 2 Results and Discussion

### 2.1 Characterization of TiO_2_ Nanosheets and Hybrid Dielectric Films

**Figure 2:**
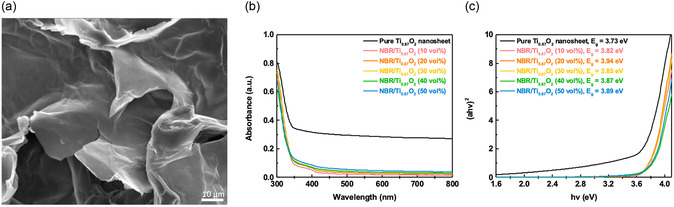
SEM analysis of a) TiO_2_ nanosheets; b) the UV–vis absorption spectra and c) (*ahv*)^2^ versus energy gap of nanocomposite NBR/TiO_2_‐*x* (*x* = 10, 20, 30, 40, 50 vol%) films.

**Figure 3:**
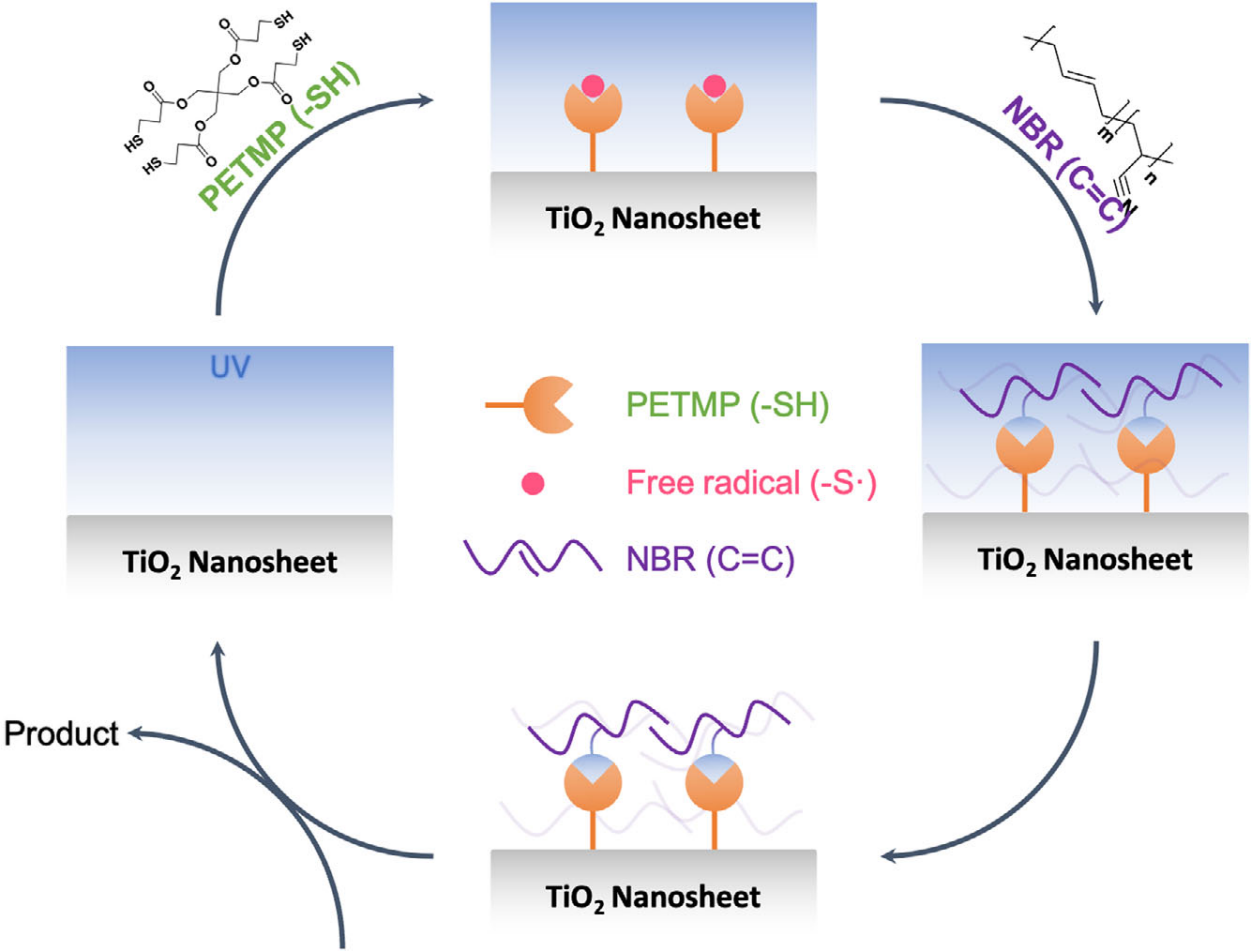
Thiol‐ene radical reaction scheme between TiO_2_ nanosheets and NBR. The orange group is the thiol functional group (—SH) of PETMP; the purple chain is NBR; and the pink dots are free radicals.

**Figure** **2**a shows the sheetlike structure of TiO_2_ nanosheets under scanning electron microscope (SEM), which is similar to the previously reported results.^[^ ^35^, ^43^ ^]^ In Figure 2b, we present the ultraviolet–visible (UV–vis) absorption spectra of TiO_2_ nanosheets and NBR/TiO_2_‐*x* (*x* = 10, 20, 30, 40, 50 vol%) composite films. The energy gap (*E*_g_) increases from 3.82 eV for NBR/TiO_2_‐10 to 3.89 eV for NBR/TiO_2_‐50, as shown in Figure 2c. The results show that TiO_2_ nanosheets in nanocomposite dielectrics are affected. It is shown that the synergistic effect resulting from the doping of 2D materials in the dielectric films enhances the insulating properties. This behavior is attributed to the thiol‐ene radical reaction induced by TiO_2_ nanosheets, NBR, the cross‐linking agent pentaerythritol tetrakis(3‐mercaptopropionate) (PETMP), and the photoinitiator diphenyl(2,4,6‐trimethylbenzoyl)phosphine oxide (TPO). **Figure** **3** illustrates the cross‐linking reaction scheme. Under UV irradiation, the free radicals generated by TiO_2_ nanosheets and TPO tend to attack the thiol groups (—SH) of PETMP, and the resulting intermediates further react with alkene (—CH=CH—) provided by NBR as the reactive sites for cross‐linking.^[^ ^44^, ^45^ ^]^ Increasing the concentration of nanosheets led to a more complete photocured structure and significantly improved the insulating properties for the nanocomposite films.

### 2.2 Morphological Characterization of Dielectric Films

**Figure 4:**
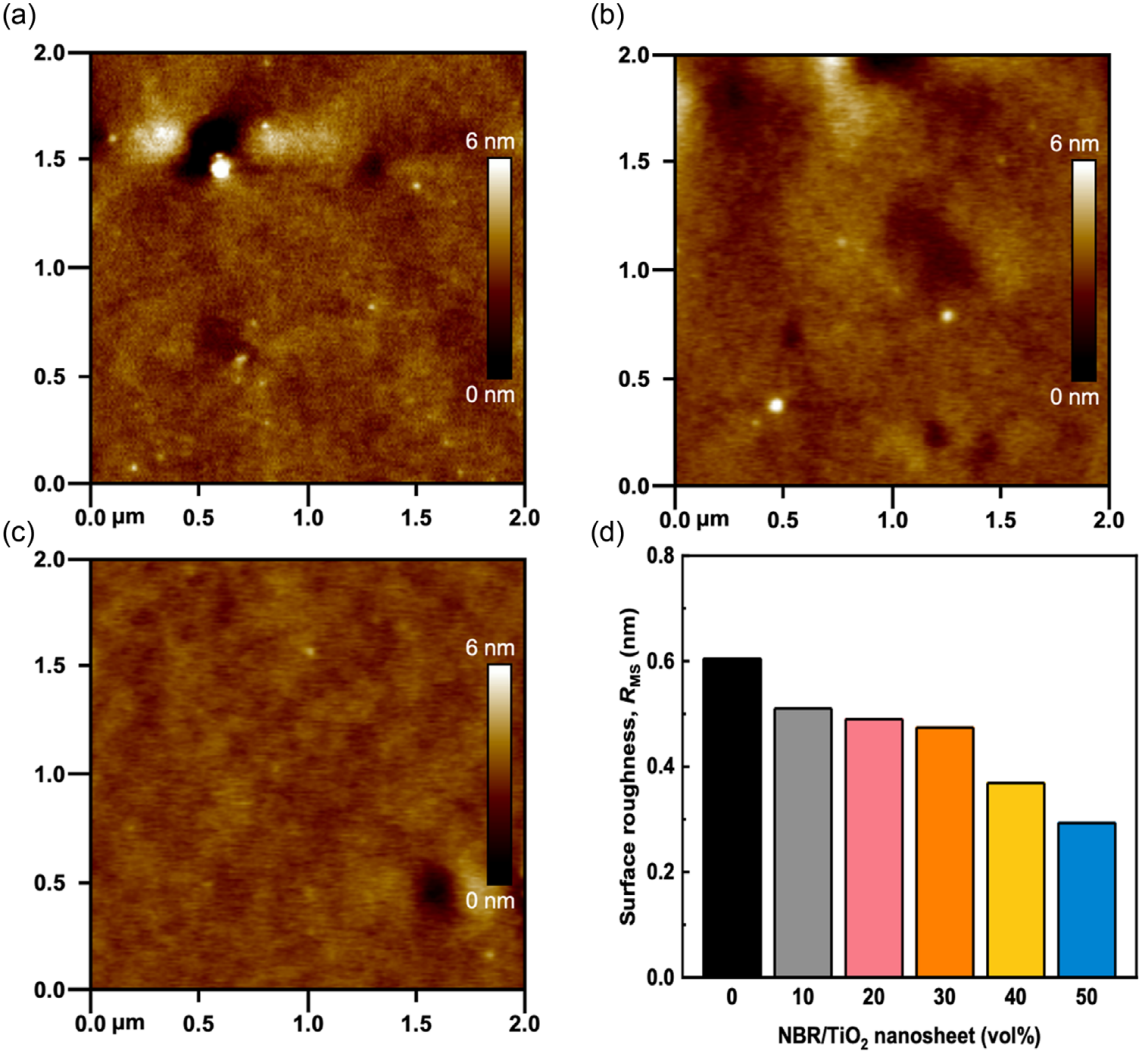
AFM height images of nanocomposite films corresponding to a) pristine NBR, b) NBR/TiO_2_‐30, and c) NBR/TiO_2_‐50. d) Summary of surface roughness of nanocomposite dielectric films.

We first explored the surface morphology changes and phase separation behavior of the nanocomposite dielectric films by atomic force microscopy (AFM). As shown in **Figure** **4**a, the roughness of the pristine NBR film, which was used for photo‐cross‐linking, was 0.604 nm. Figure 4b,c shows the hybrid films with different volume fractions. The nanocomposite films all exhibit similar smooth surfaces with roughnesses of 0.51, 0.49, 0.47, 0.37, and 0.29 nm, corresponding to NBR/TiO_2_‐*x* (*x* = 10, 20, 30, 40, 50) as shown in Figure 4d. The AFM images of the remaining nanocomposite films are shown in Figure S1, Supporting Information. The doping concentration of TiO_2_ nanosheets affects the surface morphology and roughness of the nanocomposite dielectrics. The higher the content of TiO_2_ nanosheets, the smoother the surface of the films. In contrast, some aggregates were observed in the NBR film as shown in Figure S2a, Supporting Information. Figure S2b–f, Supporting Information, reveals that the introduction of TiO_2_ makes the film surface smoother. When the volume fraction reached 30%, the film surface improved significantly and a few aggregates appeared. Additionally, we did not find any sheetlike structure similar to TiO_2_ nanosheets on the film surface. Therefore, we conclude that TiO_2_ nanosheets cross‐linked with NBR. TiO_2_ nanosheets were completely encapsulated in the dielectric films.^[^ ^40^, ^46^ ^]^ As the concentration of TiO_2_ nanosheet increases, the dopant is uniformly dispersed in the polymer, forming a complete cross‐linking of the dielectrics. It also improves surface roughness and reduces aggregation. As shown in Figure S3, Supporting Information, we demonstrate the contact angle measurements of NBR and NBR/TiO_2_‐*x* (*x* = 10, 20, 30, 40, 50 vol%) nanocomposite films. The contact angle increased from 80.8° to 87.8°. The increase in TiO_2_ doping concentration enhanced the cross‐linking between NBR and TiO_2_, resulting in TiO_2_ nanosheets encapsulated by NBR. The result showed that the surface hydrophobicity of the nanocomposite dielectric films was enhanced with the increase of TiO_2_ doping concentration. This variation in the hydrophilic/hydrophobic properties of the nanocomposite film surfaces is consistent with the trend observed by Tengfei S. et al.^[^ ^46^ ^]^ These results elucidate the contribution of TiO_2_ nanosheets to NBR in the cross‐linking reaction. The surface of the dielectric was enhanced not only by the thiol‐ene radical reaction between TiO_2_ nanosheets and NBR, but also by the tuning of the surface hydrophobicity, which reduced interfacial defects when the conjugated polymers form high‐quality films in the solution process. Furthermore, Figure S4, Supporting Information, shows the variation in absorbance of different nanocomposite dielectric films. The films show strong absorption IR peak at 8.80, 9.69, and 12.46 μm. The thermal emission is highly dependent on the IR absorption.^[^ ^47^ ^]^ Therefore, these IR characteristics reveal the potential of nanocomposite dielectric films to improve the thermal emission behavior of integrated electronics.

### 2.3 Insulating Properties of Nanocomposite Dielectrics

**Figure 5:**
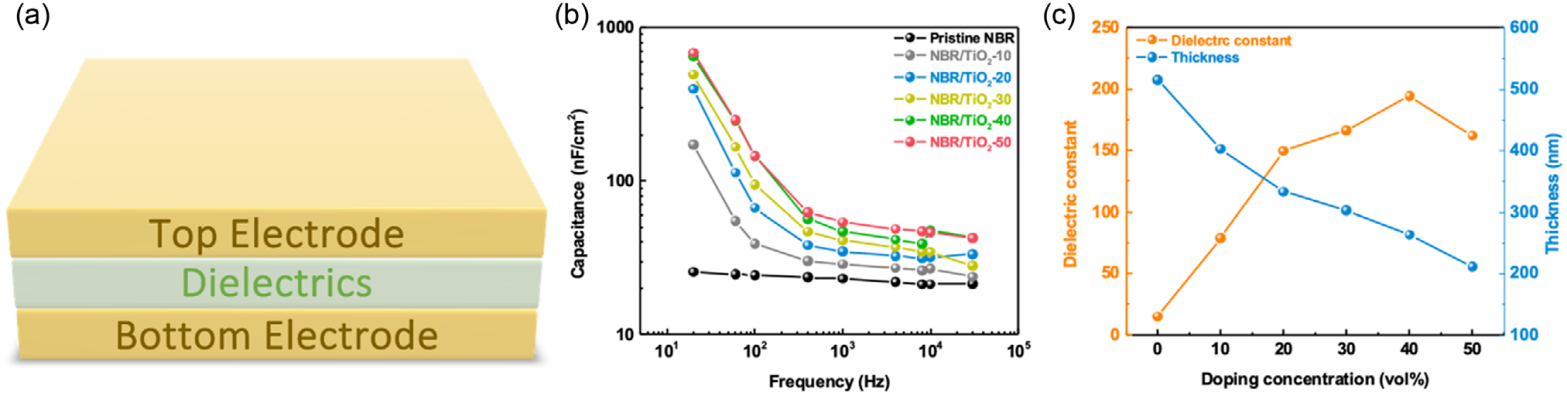
a) Schematic of metal–insulator–metal (MIM) capacitor. b) Comparison of capacitance of nanocomposite dielectric films at different frequencies. c) Effect of doped TiO_2_ nanosheets on permittivity and thickness.

The capacitance of these nanocomposite films was assembled in a two‐terminal metal–insulator–metal (MIM) capacitor structure (**Figure** **5**a) to measure insulator properties. A bare wafer without a silicon dioxide layer on the surface was used as the bottom electrode, and gold (80 nm) was deposited onto the surface of the hybrid dielectric film by thermal evaporation as the top electrode. Figure 5b illustrates the capacitive response to nanocomposite dielectrics with different doping concentrations. The dielectric capacitance is strongly dependent on the doping concentration and is frequency dependent. The limited polarization response time within the dielectrics causes the capacitance to decrease with frequency.^[^ ^12^, ^16^ ^]^ The capacitance values of NBR films and NBR/TiO_2_‐*x* (*x* = 10, 20, 30, 40, 50 vol%) nanocomposite films measured at 20 Hz were 25.61, 173.12, 397.07, 494.55, 647.84, and 684.67 nF cm^2^, respectively. The dielectric constants of the pristine NBR film were found to be in agreement with the reported values.^[^ ^17^ ^]^ The NBR film shows potential as a high‐k dielectric material and it is observed that the nitrile group is not affected by the dipole polarization time response during frequency changes. The capacitance remains stable even at 10 kHz frequency. However, we observed a two‐level capacitance characteristic in NBR/TiO_2_ nanocomposite dielectrics. This phenomenon is attributed to the sharp change in capacitance before 1 kHz frequency due to ferroelectric polarization and titanium ion shift.^[^ ^48^, ^49^ ^]^ The concentration‐dependent capacitance enhancement of the nanocomposite film after 1 kHz is attributed to the polarization properties of the TiO_2_ nanosheets themselves affecting the nanocomposite dielectrics. When we doped a large amount of TiO_2_ nanosheets (more than 30 vol%), the growth of the relative dielectric constant of the nanocomposite film gradually saturated as shown in Figure 5c, which also represents the relative dielectric constant of the nanocomposite films doped with different volume fractions and thicknesses. The relative dielectric constants of the nanocomposite dielectric films are 14.96, 78.57, 149.49, 166.14, 194.22, and 161.98 corresponding to NBR and NBR/TiO_2_‐*x* (*x* = 10, 20, 30, 40, 50 vol%), respectively. The nanocomposite dielectric films we prepared exhibited a high dielectric constant of 194.22, higher than the previous dielectric constants of 3.27 and 43.8 for the hybrid media using PVA and PP with TiO_2_ nanosheets, respectively.^[^ ^18^, ^33^ ^]^

**Figure 6:**
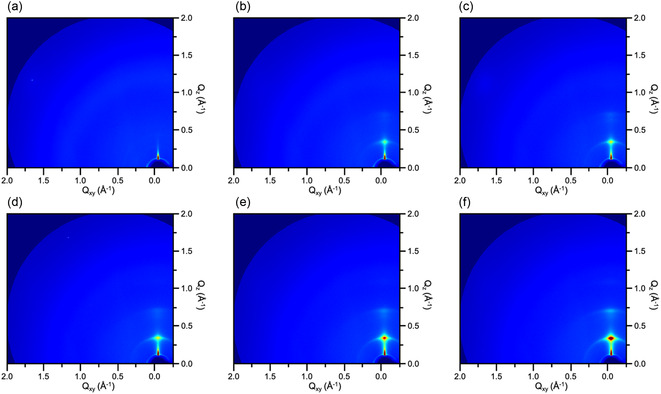
2D GIXD images of nanocomposite dielectric films corresponding to a) pristine NBR, b) NBR/TiO_2_‐10, c) NBR/TiO_2_‐20, d) NBR/TiO_2_‐30, e) NBR/TiO_2_‐40, and f) NBR/TiO_2_‐50.

Furthermore, we explored the breakdown field strength of NBR and NBR/TiO_2_‐*x* (*x* = 10, 20, 30, 40, 50 vol%) films. As shown in Figure S5, Supporting Information, the addition of TiO_2_ nanosheets improves the dielectric strength of the nanocomposite dielectrics and is proportional to the addition concentration. This increase is attributed to more complete cross‐linking of the dielectric films. Furthermore, we observed a linear decrease in the dielectric film thickness, which was 514, 402, 332, 302, 262, and 211 nm for NBR and NBR/TiO_2−_ *x* (*x* = 10, 20, 30, 40, 50 vol%), respectively. The reason for the decrease in the film thickness was that we added solution‐dispersed TiO_2_ nanosheets to the NBR solution at a controlled volume ratio, thus reducing the overall concentration of the NBR solution during the doping process. However, Equation (1) shows that film thickness affects the relative dielectric constant.

(1) $$C=\frac{\varepsilon_{0}\varepsilon A}{d}$$

where *C* is the capacitance of the dielectric, *ε*_0_ and *ε* are the vacuum and relative permittivity, respectively, *A* is the area where the top and bottom electrodes overlap with the dielectric film, and *d* is the aathickness of the dielectric film. When the thickness of the dielectric film is reduced, the capacitance value of NBR/TiO_2_‐50 increases only slightly. The results indicate that NBR/TiO_2_‐50 has a lower dielectric constant compared to the NBR/TiO_2_‐40. To further elucidate the effect of elastomer‐doped TiO_2_ nanosheets on the capacitance values, we performed crystal structure analysis of the nanocomposite dielectric films using GIXD. As shown in **Figure** **6**a, only the NBR film did not exhibit any crystal signals. Nevertheless, the nanocomposite dielectric films showed (100) and (200) peaks in the out‐of‐plane direction with increasing doping concentration as shown in Figure 6b–f. The intensity of the crystal signals is enhanced with increasing volume fraction of TiO_2_ nanosheets. Therefore, the crystal stacking generated in the out‐of‐plane direction further enhances the polarization characteristics of the nanocomposite dielectric films. We also show the transfer curves of PDPP‐TT OFETs based on nanocomposite dielectrics in Figure 8a,c and S6, Supporting Information. The PDPP‐TT OFET based on NBR/TiO_2_‐30 shows the best electric performance. We will discuss the electrical properties of OFETs in detail in Section 2.5.

### 2.4 Stretchability and Electrical Properties of Dielectrics

**Figure 7:**
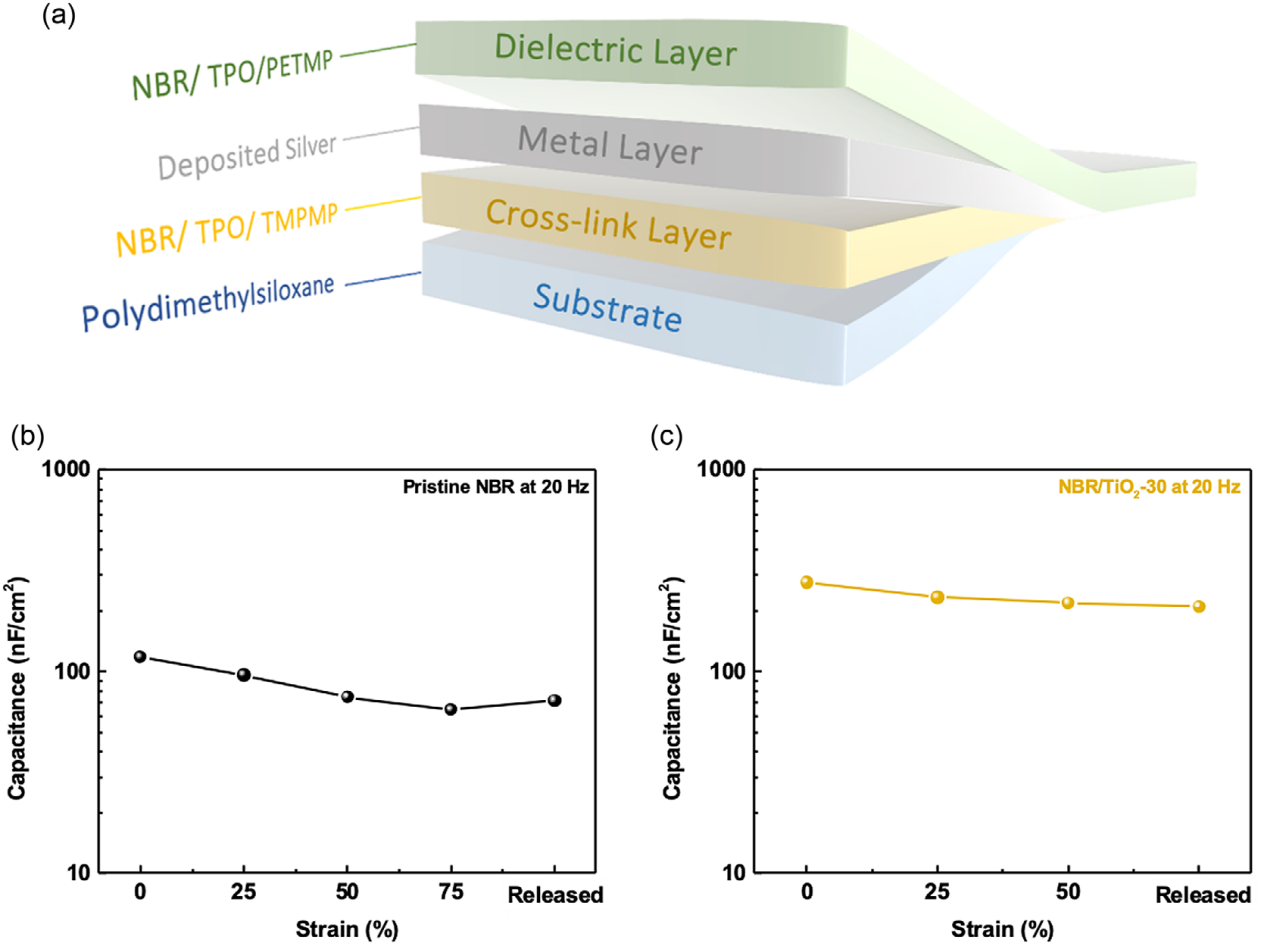
a) Schematic of our fabricated stretchable metal–insulator–metal (MIM) capacitor. Capacitance of nanocomposite dielectric films, b) pristine NBR, and c) NBR/TiO_2_‐30, at different strains at 20 Hz.

**Figure** **7**a shows a schematic of a stretchable MIM capacitor. A photo‐cross‐linked NBR‐based film was coated with a stretchable PDMS substrate as a conductive cross‐linking layer, and silver (80 nm) was thermally deposited into the polymer structure to achieve good electrical conductivity. A nanocomposite dielectric film is coated on the conductive cross‐linked layer. Then, we thermally deposited the gold (80 nm) as the top electrode to form a stretchable MIM capacitor. Figure 7b,c shows the capacitance values of NBR and NBR/TiO_2_‐30 dielectric films under different tensile strains. When the capacitor was subjected to tensile strain, the capacitance values of the NBR film were 117.87, 95.97, 74.79, and 64.70 nF cm^−2^ corresponding to 0%, 25%, 50%, and 75% strain at 20 Hz, respectively. The capacitance values of NBR/TiO_2_‐30 dielectric film measured at the same frequency were 276.95, 233.42, and 218.64 nF cm^−2^ at 0%, 25%, and 50% strains, respectively. However, the nanocomposite dielectric films did not show capacitance decay under tensile strains as a result of stress changes, indicating that the addition of inorganic materials to the polymers provides good intrinsic tensile properties through photo‐cross‐linking. We further measured the mechanical properties of the nanocomposite films, as presented in Figure S7, Supporting Information. The results showed that the maximum strain of the nanocomposite films could reach 130%. The modulus also increased slightly with the increase of TiO_2_ doping concentration. This result is attributed to the participation of TiO_2_ nanosheets in the cross‐link reaction, which makes the structure more stable and able to release the stress through the polymer network structure in the nanocomposite film under the influence of tensile strain. In Figure S8a,b, Supporting Information, we observed the top electrode of the capacitor at tensile strains of 0% and 100%, respectively, using an optical microscope, and the results show that the original smooth and continuous morphology is transformed into a discontinuous electrode with wrinkles after high‐intensity stretching. The results show that the capacitance of the NBR/TiO_2_‐30 dielectric film can only be detected at a strain of 50%. Finally, we observed that the surface of the top electrode maintained the wrinkled morphology after release as shown in Figure S8c, Supporting Information. This indicates that the surface was wrinkled due to tensile stress. Thus, the results suggest that the generation of these microcracks affects the conductivity of the electrodes, which not only leads to the inability to probe the insulating properties of the nanocomposite dielectric films at high tensile strains, but also the capacitance values in the released state are slightly lower than the initial capacitance values of the NBR/TiO_2_‐30 dielectric film.

### 2.5 Electrical Properties of OFETs Based on Nanocomposite Dielectric Films

**Figure 8:**
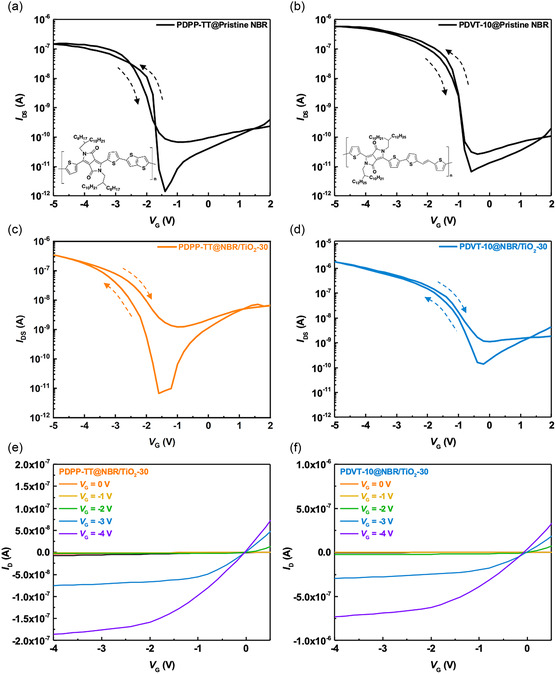
Transfer characteristics of a,c) PDPP‐TT and b,d) PDVT‐10 OFETs based on a,b) pristine NBR and c,d) NBR/TiO_2_‐30 dielectrics. Output curves of e) PDPP‐TT and f) PDVT‐10 OFETs based on the NBR/TiO_2_‐30 dielectric.

To efficiently investigate how organic/inorganic hybrid nanocomposite dielectrics affect polymer FETs, we used two high‐performance *p*‐type conjugated polymers, PDPP‐TT and PDVT‐10, whose chemical structures are shown in Figure 1. In **Figure** **8**a,b, the transfer characteristic curves of PDPP‐TT and PDVT‐10 are based on NBR films. Figure 8c,d shows the transfer curves of OFETs based on NBR/TiO_2_‐30. Subsequently, we evaluated the performance of these devices based on capacitance at 1 kHz and showed average mobilities of 0.256, 0.303, 0.132, and 0.455 cm^2^ V^−1^ s^−1^ corresponding to the device sequences shown in Figure 8a–d. We summarize the electrical characteristics of PDPP‐TT OFETs based on the NBR/TiO_2_‐*x* (*x* = 10, 20, 40, 50 vol%), as shown in Table S1, Supporting Information. These results clearly define the linear and saturation regions of the polymer FETs (Figure 8e,f). In this work, the capacitance values of nanocomposite dielectrics vary significantly between 20 Hz and 1 kHz. To avoid the polarization characteristics that lead to an underestimation of the quality factor of OFETs, we use transconductance (*g*_m_) to define the device performance. The current on/off ratios of OFETs with nanocomposite films as dielectric layers are 10^4^–10^5^. Therefore, the *g*_m_ of the NBR/TiO_2_‐30‐based PDPP‐TT OFET is as high as 0.202 μS, whereas the *g*_m_ is as high as 0.808 μS when we use PDVT‐10 as the active channel. Furthermore, we modulated the scan speed of the PDPP‐TT OFET based on NBR/TiO_2_‐30 dielectric from 37 to 245 mV s^−1^ to examine the effect of ferroelectric polarization on the OFET. As shown in Figure S9, Supporting Information, the transmission characteristics of the OFETs are related to the scan speed. As the scan speed changes from slow to fast, the backward sweep current moves from right to left. This observation is consistent with previous reports on the FeFETs.^[^ ^50^, ^51^ ^]^ The reason for this phenomenon is that the scan speed affects the polarization response time. At slower scan rater, the NBR/TiO_2_ nanocomposite dielectric film has enough time for polarization response. Conversely, at faster scan rates, the nanocomposite dielectrics did not have enough time to achieve full polarization, thus affecting the performance of OFETs.

In addition, we also observed an interesting current hysteresis behavior in the transfer curves. Both PDPP‐TT and PDVT‐10 OFETs composed of pristine NBR exhibit low backward sweep currents. However, OFETs based on the NBR/TiO_2_‐30 dielectric show higher backward sweep currents. This shift in hysteresis characteristics suggests that the incorporation of TiO_2_ nanosheets induces a ferroelectric‐like material polarization, which affects the hysteresis characteristics. There are several possible reasons to explain the change in the backward sweep current. First, there is a competitive interaction between the attractiveness of TiO_2_ nanosheets and the polarity of the nitrile (—CN) groups, which leads to a charge‐trapping behavior that further reduces the backward sweep current in the hysteresis curve. Similarly, the expansion of the doping volume fraction of TiO_2_ nanosheets in the doped film promotes complete cross‐linking based on the thiol‐ene click chemistry, which reduces the dielectric film thickness and surface roughness. This inhibits the formation of interface traps and enhances the ferroelectricity due to the increased content of TiO_2_ nanosheets.

**Figure 9:**
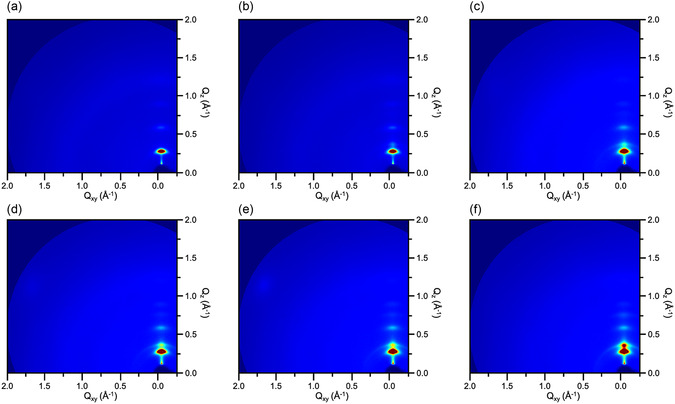
2D GIXD images of PDPP‐TT OFETs based on a) pristine NBR, b) NBR/TiO_2_‐10, c) NBR/TiO_2_‐20, d) NBR/TiO_2_‐30, e) NBR/TiO_2_‐40, and f) NBR/TiO_2_‐50 dielectric films, respectively.

Next, we analyzed the crystal arrangement of conjugated polymers on nanocomposite dielectrics using GIXD. In **Figure** **9**a, only the crystal signal of PDPP‐TT is observed in the out‐of‐plane direction. In contrast, on the OFET based on the nanocomposite dielectric film shown in Figure 9b–f, we observed crystal signals corresponding to the (100) and (200) peaks of the TiO_2_ nanosheets in the out‐of‐plane direction. These signals are enhanced with increasing doping concentration. This result further suggests that the incorporation of more ferroelectric TiO_2_ nanosheets into the dielectric layer not only facilitates the complete cross‐linking of the polymer, but also reduces the generation of interface traps between the dielectric layer and the active channel. In addition, the layer‐by‐layer stacking of nanosheets in the out‐of‐plane direction enhances the ferroelectric polarization property, which leads to the increase of backward sweep current in OFET.

## 3 Conclusions

This study describes the potential of 2D materials doped with dielectric layers for applications in electronic and stretchable devices. TiO_2_ nanosheets and NBR form a cross‐linked network through a thiol‐ene reaction to form nanocomposite films, which results in smoother film surface and exhibits high capacitance characteristics. Polymer FETs utilizing nanocomposite dielectrics demonstrate higher transconductance, on/off ratio, and lower threshold voltage. Furthermore, controlling the doping concentration of TiO_2_ nanosheets show different electrical characteristics. While the doping concentration is increased by more than 30 vol%, the current hysteresis behavior changes from charge trapping to polarization characteristics. Additionally, fully stretchable capacitors based on nanocomposite dielectric films were tested for insulating properties under different strains. The stretchable capacitor maintains excellent dielectric properties even at a strain 50%. Thus, this work demonstrates the great potential of incorporating 2D materials into polymer films as dielectric layers for stretchable devices.

## 4 Experimental Section

4.1

4.1.1

##### Device Fabrication and Characterization

We prepared the NBR solution by dissolving poly(acrylonitrile*‐co*‐butadiene) in chlorobenzene (CB) at a concentration of 50 mg mL^−1^ with PETMP and TPO at a concentration of 40 mg mL^−1^ as cross‐linker and initiator. The volumetric ratio of NBR:TPO:PETMP was 32:1:1. Titania nanosheets (Ti_0.87_O_2_) were dispersed in CB by sonication for 30 mins. The dispersed Ti_0.87_O_2_ was then mixed into the NBR solution at volume percentages of 10, 20, 30, 40, and 50. The dielectric films were spin‐coated on bare silicon substrates at a spin rate of 1000 rpm for 60 s. The films were further thermally annealed at 60 °C for 10 min and UV cured (365 nm) for 5 min. Subsequently, semiconducting polymers (PDPP‐TT and PDVT‐10, 5 mg mL^−1^ in CB) were spin‐coated onto NBR dielectric films at 1000 rpm for 60 s and thermally annealed at 150 °C for 1 h. OFET adopted a bottom‐gate top‐contact device structure. The source and drain electrodes were 80 nm thick gold electrodes thermally evaporated on the polymer film through a shadow mask with the channel length (*L*) and width (*W*) of 50 and 1000 μm, respectively.

Stretchable capacitors were fabricated as follows: polydimethylsiloxane (PDMS) was prepared as a stretchable substrate using a base polymer and curing agent in a ratio of 20:1. The cross‐link film was prepared by spin‐coating the NBR solution with Trimethylolpropane tris(3‐mercaptopropionate (TMPMP) and TPO (volumetric ratio = 32:1:1) onto the PDMS substrate at 3000 rpm for 10 s, followed by thermal annealing at 50 °C for 10 min and UV cured for 5 min. An 80 nm thick Ag electrode was then thermally evaporated on the PDMS to serve as the bottom electrode. The stretchable dielectric films were spin‐coated onto the silver‐evaporated PDMS and then thermally annealed at 60 °C for 10 min and UV cured for 5 min. Afterward, an 80 nm thick Au electrode was evaporated on the stretchable dielectric film to prepare a two‐terminal MIM capacitor. The electrical properties of OFETs were measured using a Keithley 2634B semiconductor parameter analyzer in a nitrogen‐filled glove box. The insulator properties of capacitors were measured using an Agilent 4284 A Precision LCR meter.

## Conflict of Interest

The authors declare no conflict of interest.

## Author Contributions

**Qun‐Gao Chen**: Data Curation (Lead), Investigation (Lead), Methodology (Lead), Validation (Lead), and Writing—Original Draft (Lead); **Xingke Cai**: Conceptualization (Supporting) and and Supervision (Supporting); **Chu‐Chen Chueh**: Supervision (Supporting) and Writing—Review & Editing (Equal); and **Wen‐Ya Lee**: Conceptualization (Lead), Resources (Lead), Supervision (Lead), and Writing—Review & Editing (Lead).

## Supporting information

Supplementary Material

## Data Availability

The data that support the findings of this study are available from the corresponding author upon reasonable request.

## References

[1] J. C. Yang , S. Lee , B. S. Ma , J. Kim , M. Song , S. Y. Kim , D. W. Kim , T. S. Kim , S. Park , Sci. Adv. 2022, 8, eabn3863. 35648853 10.1126/sciadv.abn3863 PMC9159573

[2] C. A. Silva , J. Lv , L. Yin , I. Jeerapan , G. Innocenzi , F. Soto , Y. G. Ha , J. Wang , Adv. Funct. Mater. 2020, 30, 2002041.

[3] J. C. Yang , J. Mun , S. Y. Kwon , S. Park , Z. Bao , S. Park , Adv. Mater. 2019, 31, 1904765. 10.1002/adma.201904765 31538370

[4] M. Kaltenbrunner , T. Sekitani , J. Reeder , T. Yokota , K. Kuribara , T. Tokuhara , M. Drack , R. Schwödiauer , I. Graz , S. Bauer‐Gogonea , S. Bauer , T. Someya , Nature 2013, 499, 458. 23887430 10.1038/nature12314

[5] H. G. Park , M. Kim , H. Park , J. H. Oh , Adv. Funct. Mater. 2024, 34, 2312034.

[6] Q. Su , Q. Zou , Y. Li , Y. Chen , S.‐Y. Teng , J. T. Kelleher , R. Nith , P. Cheng , N. Li , W. Liu , Sci. Adv. 2021, 7, eabi4563. 34818045 10.1126/sciadv.abi4563 PMC8612682

[7] G. J. N. Wang , A. Gasperini , Z. A. Bao , Adv. Electron. Mater. 2018, 4, 1700429.

[8] J. Mun , Y. Ochiai , W. C. Wang , Y. Zheng , Y. Q. Zheng , H. C. Wu , N. Matsuhisa , T. Higashihara , J. B. H. Tok , Y. Yun , Z. A. Bao , Nat. Commun. 2021, 12, 3572. 34117254 10.1038/s41467-021-23798-2 PMC8196107

[9] J. Xu , H. C. Wu , J. Mun , R. Ning , W. C. Wang , G. J. N. Wang , S. Nikzad , H. P. Yan , X. D. Gu , S. C. Luo , D. S. Zhou , J. B. H. To , k , Z. N Bao , Adv. Mater. 2022, 34, 2104747. 10.1002/adma.202104747 34558121

[10] H. C. Tien , X. Li , C. J. Liu , Y. Li , M. Q. He , W. Y. Lee , Adv. Funct. Mater. 2023, 33, 2211108.

[11] W. Wang , S. Wang , R. Rastak , Y. Ochiai , S. Niu , Y. Jiang , P. K. Arunachala , Y. Zheng , J. Xu , N. Matsuhisa , Nat. Electron. 2021, 4, 143.

[12] B. H. Wang , W. Huang , L. F. Chi , M. Al‐Hashimi , T. J. Marks , A. Facchetti , Chem. Rev. 2018, 118, 5690. 29785854 10.1021/acs.chemrev.8b00045

[13] J. Y. Pei , S. L. Zhong , Y. Zhao , L. J. Yin , Q. K. Feng , L. Huang , D. F. Liu , Y. X. Zhang , Z. M. Dang , Energy Environ. Sci. 2021, 14, 5513.

[14] S. Wang , C. Yang , X. M. Li , H. Y. Jia , S. R. Liu , X. Y. Liu , T. Minari , Q. Q. Sun , J. Mater. Chem. C 2022, 10, 6196.

[15] A. Chortos , G. I. Koleilat , R. Pfattner , D. S. Kong , P. Lin , R. Nur , T. Lei , H. L. Wang , N. Liu , Y. C. Lai , M. G. Kim , J. W. Chung , S. Lee , Z. N. Bao , Adv. Mater. 2016, 28, 4441. 26179120 10.1002/adma.201501828

[16] C. Lu , W. Y. Lee , C. C. Shih , M. Y. Wen , W. C. Chen , ACS Appl. Mater. Interfaces 2017, 9, 25522. 28665108 10.1021/acsami.7b06765

[17] W. Wang , Y. Jiang , D. Zhong , Z. Zhang , S. Choudhury , J. C. Lai , H. Gong , S. Niu , X. Yan , Y. Zheng , C. C. Shih , R. Ning , Q. Lin , D. Li , Y. H. Kim , J. Kim , Y. X. Wang , C. Zhao , C. Xu , X. Ji , Y. Nishio , H. Lyu , J. B. H. Tok , Z. Bao , Science 2023, 380, 735. 37200416 10.1126/science.ade0086

[18] M. Jang , S. Y. Park , S. K. Kim , D. Jung , W. Song , S. Myung , S. S. Lee , D. H. Yoon , K. S. An , Small 2021, 17, 2007213. 10.1002/smll.202007213 33719185

[19] M. Osada , Y. Ebina , H. Funakubo , S. Yokoyama , T. Kiguchi , K. Takada , T. Sasaki , Adv. Mater. 2006, 18, 1023.

[20] K. Akatsuka , M. Haga , Y. Ebina , M. Osada , K. Fukuda , T. Sasaki , ACS Nano 2009, 3, 1097. 19402657 10.1021/nn900104u

[21] M. Osada , T. Sasaki , APL Mater. 2019, 7, 120902.

[22] G. L. Xiang , T. Y. Li , J. Zhuang , X. Wang , Chem. Commun. 2010, 46, 6801. 10.1039/c0cc02327b 20730205

[23] Q. Guo , C. Zhou , Z. Ma , X. Yang , Adv. Mater. 2019, 31, 1901997. 10.1002/adma.201901997 31423680

[24] A. Meng , L. Zhang , B. Cheng , J. Yu , Adv. Mater. 2019, 31, 1807660. 10.1002/adma.201807660 31148244

[25] B. Tatykayev , B. Chouchene , L. Balan , T. Gries , G. Medjahdi , E. Girot , B. Uralbekov , R. Schneider , Nanomaterials 2020, 10, 1387. 32708780 10.3390/nano10071387 PMC7407120

[26] P. H. Wöbkenberg , T. Ishwara , J. Nelson , D. D. C. Bradley , S. A. Haque , T. D. Anthopoulos , Appl. Phys. Lett. 2010, 96, 082116.

[27] X. Cai , N. Sakai , T. C. Ozawa , A. Funatsu , R. Ma , Y. Ebina , T. Sasaki , ACS Appl. Mater. Interfaces 2015, 7, 11436. 25945510 10.1021/acsami.5b02107

[28] S. Sekizaki , M. Osadab , K. Nagashio , Nanoscale 2017, 9, 6471. 28466951 10.1039/c7nr01305a

[29] X. Cai , L. Yin , N. Sakai , D. Liu , C. Teng , Y. Ebina , R. Ma , T. Sasaki , ACS Appl. Nano Mater. 2019, 2, 6378.

[30] A. Moudgil , S. Singh , N. Mishra , P. Mishra , S. Das , Adv. Mater. Technol. 2020, 5, 1900615.

[31] T. Gakhar , A. Hazra , Measurement 2021, 182, 109721.

[32] N. N. Rabin , S. Ida , M. R. Karim , M. S. Islam , R. Ohtani , M. Nakamura , M. Koinuma , L. F. Lindoy , S. Hayami , ACS Omega 2018, 3, 2074. 31458516 10.1021/acsomega.7b01764 PMC6641247

[33] W. Tian , F. Yan , C. Cai , Z. Wu , C. Zhang , T. Yin , S. Lao , L. Hu , J. Adv. Ceram. 2021, 10, 368.

[34] W. Ma , K. Yang , C. Zhou , H. Li , Ceram. Int. 2022, 48, 10447.

[35] P. Xiong , X. Zhang , F. Zhang , D. Yi , J. Zhang , B. Sun , H. Tian , D. Shanmukaraj , T. Rojo , M. Armand , R. Ma , T. Sasaki , G. Wang , ACS Nano 2018, 12, 12337. 30427658 10.1021/acsnano.8b06206

[36] R. S. Dubey , S. R. Jadkar , A. B. Bhorde , ACS Omega 2021, 6, 3470. 33585733 10.1021/acsomega.0c01614 PMC7876674

[37] Y. Tian , J. J. G. Moreno , Z. Lu , L. Li , M. Hu , D. Liu , Z. Jian , X. Cai , Chem. Eng. J. 2021, 407, 127198.

[38] G. Luo , D. Liu , J. Zhao , A. Hussain , W. Raza , Y. Wu , F. Liu , X. Cai , Small 2023, 19, 2206176. 10.1002/smll.202206176 36587971

[39] R. Tedja , A. H. Soeriyadi , M. R. Whittaker , M. Lim , C. Marquis , C. Boyer , T. P. Davis , R. Amal , Polym. Chem. 2012, 3, 2743.

[40] E. Schechtel , Y. Yan , X. Xu , Y. Cang , W. Tremel , Z. Wang , B. Li , G. Fytas , J. Phys. Chem. C 2017, 121, 25568. 10.1021/acs.jpcc.7b08425 PMC5941249 29755637

[41] L. Xiong , F. Zhan , H. Liang , L. Chen , D. Lan , J. Mater. Sci. 2018, 53, 2594.

[42] Z. Li , X. Xing , J. Zhang , M. Li , Q. Y. Zhang , Microporous Mesoporous Mater. 2020, 291, 109696.

[43] M. Leng , Y. Chen , J. Xue , Nanoscale 2014, 6, 8531. 24958361 10.1039/c4nr00946k

[44] V. T. Bhat , P. A. Duspara , S. Seo , N. S. B. Abu Bakar , M. F. Greaney , Chem. Commun. 2015, 51, 4383. 10.1039/c4cc09987g 25675845

[45] C. E. Hoyle , C. N. Bowman , Angew. Chem., Int. Ed. 2010, 49, 1540. 10.1002/anie.200903924 20166107

[46] T. Shao , W. Zhen , J. Chen , Polym. Adv. Technol. 2023, 34, 1087.

[47] Z. Wang , Y. Wang , Y. Liu , J. Xu , L. Guo , Y. Zhou , J. Ouyang , J. Dai , Curr. Appl. Phys. 2011, 11, 1405.

[48] S. Kalaiarasi , M. Jose , Appl. Phys. A: Mater. Sci. Process. 2017, 123, 512.

[49] H. Sun , Q. Wang , Y. Li , Y. F. Lin , Y. Wang , Y. Yin , Y. Xu , C. Liu , K. Tsukagoshi , L. Pan , X. Wang , Z. Hu , Y. Shi , Sci. Rep. 2014, 4, 7227. 25428665 10.1038/srep07227 PMC4245676

[50] T. Hao , B. Zeng , Z. Sun , Z. Wang , Y. Jiang , Q. Peng , S. Zheng , Y. Zhou , M. Liao , APL Mater. 2024, 12, 011108.

[51] A. Kumar , X. Song , M. M. De Souza , MRS Adv. 2021, 6, 540.

