# Supplementary material for: Photo‐Curable Stretchable High‐k Polymer/TiO2 Nanosheet Hybrid Dielectrics for Field‐Effect Transistors
Source: Small Sci. 2024 Sep 10;4(10):2400197. doi: 10.1002/smsc.202400197 (PMC11935061; doi:10.1002/smsc.202400197)
Supplement: Supplementary file 1 — Supplementary Material [file SMSC-4-2400197-s001.pdf]

Supporting Information

Photo-Curable Stretchable High-k Polymer/TiO_2 Nanosheet Hybrid Dielectrics for Field-Effect Transistors

Qun-Gao Chen,[a] *Xingke Cai,*[b,*] *Chu-Chen Chueh,*[c,*] *Wen-Ya Lee*[a,*]

[a] Q.-G. Chen and Prof. W.-Y. Lee
Department of Chemical Engineering and Biotechnology, National Taipei University of Technology, Taipei 106, Taiwan

[b] Prof. X. Cai
Institute for Advanced Study, Shenzhen University, Shenzhen 518060, P. R. China

[c] Prof. C.-C. Chueh
Department of Chemical Engineering, National Taiwan University, Taipei 10617, Taiwan

***Corresponding author.** E-mail: wenyalee@mail.ntut.edu.tw; cchueh@ntu.edu.tw; cai.xingke@szu.edu.cn

Keywords: Dielectric layer; TiO_2 nanosheet; nanocomposites; polymer field-effect transistor; stretchability; crosslinking

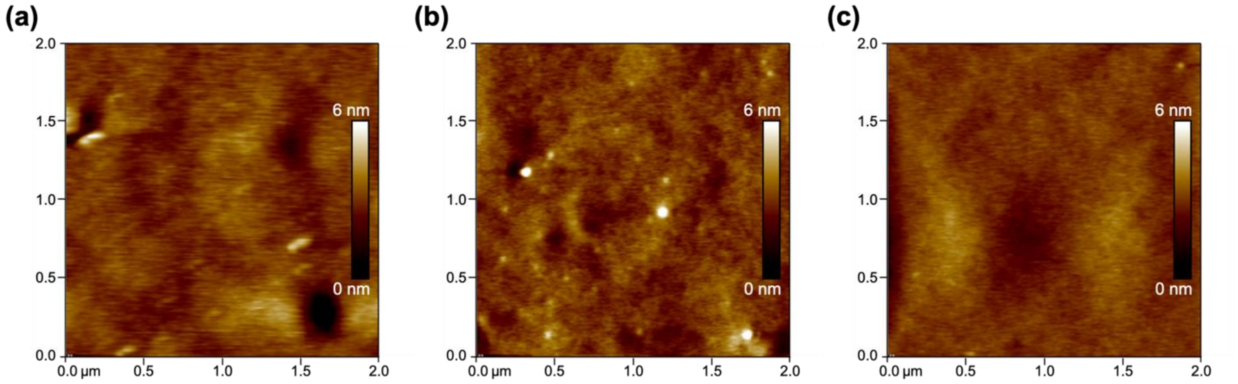

Figure S1. AFM height images of nanocomposite films corresponding to (a) NBR/TiO_2-10, (b) NBR/TiO_2-20, and (c) NBR/TiO_2-40.

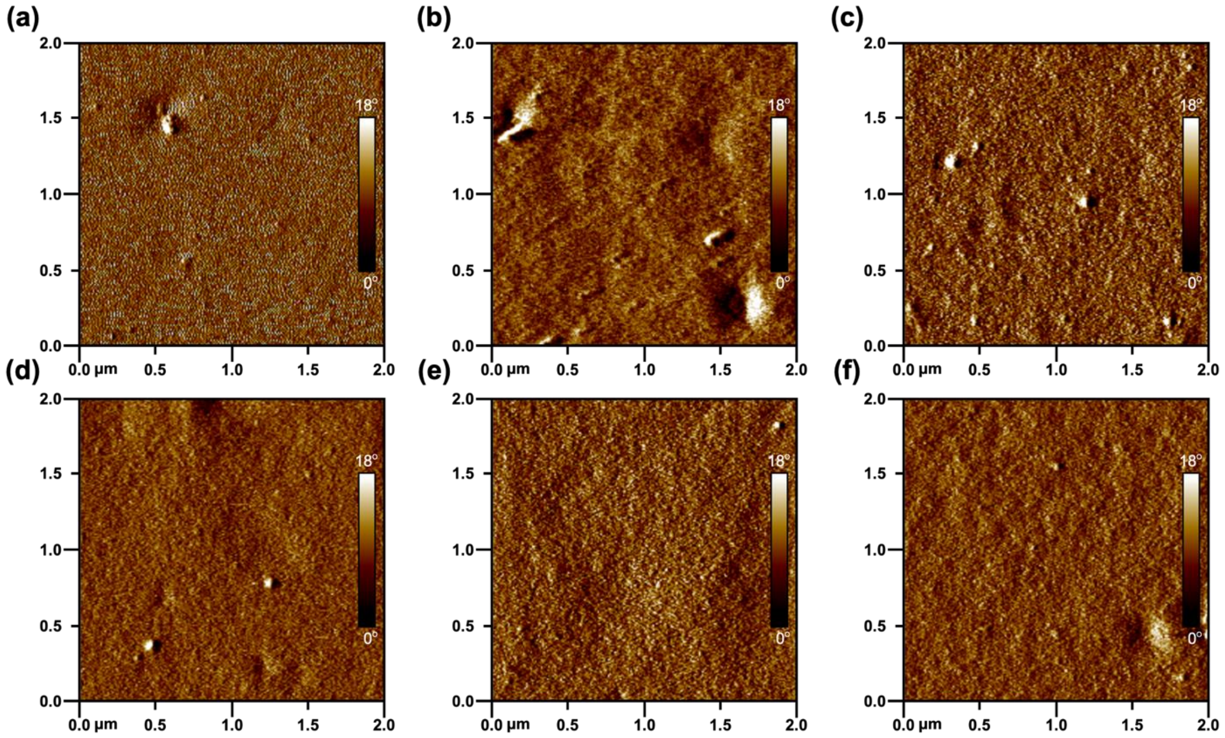

Figure S2. AFM phase images showing aggregation of (a) pristine NBR, (b) NBR/TiO_2-10, (c) NBR/TiO_2-20, (d) NBR/TiO_2-30, (e) NBR/TiO_2-40, and (f) NBR/TiO_2-50.

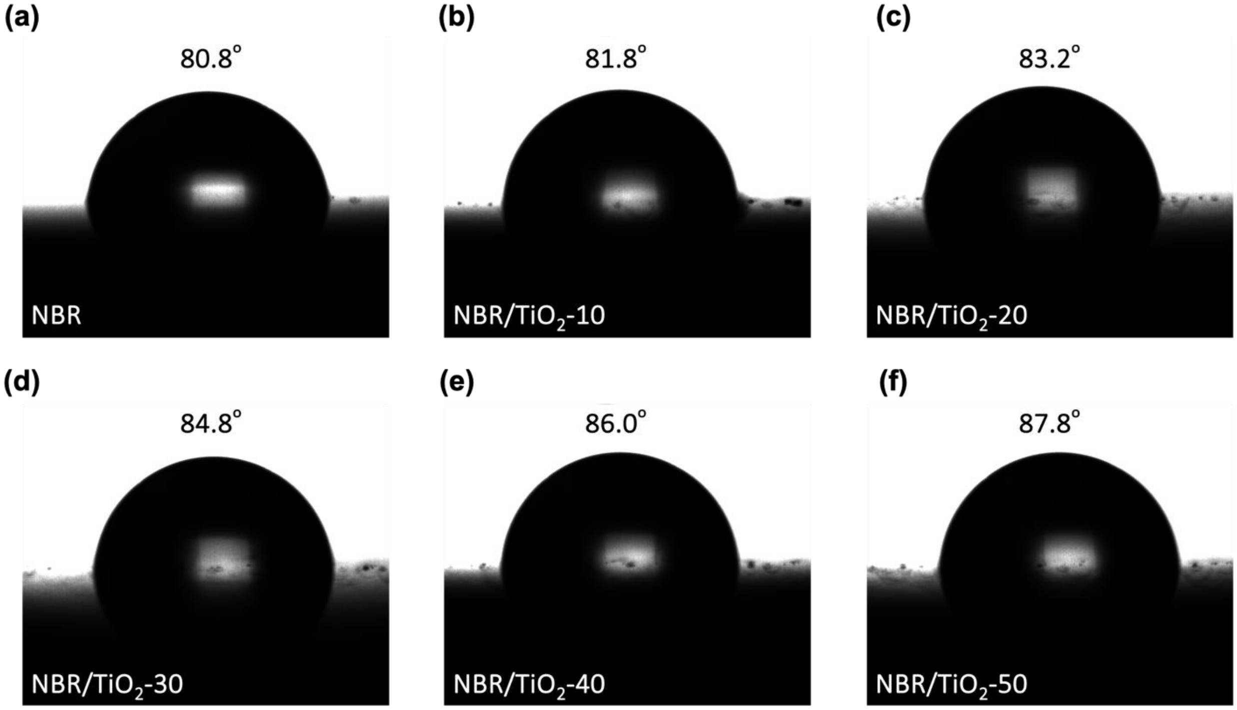

Figure S3. Water contact angles of (a) NBR, (b) NBR/TiO_2-10, (c) NBR/TiO_2-20, (d) NBR/TiO_2-30, (e) NBR/TiO_2-40, and (f) NBR/TiO_2-50 to investigate the surface changes after doping TiO_2 nanosheets.

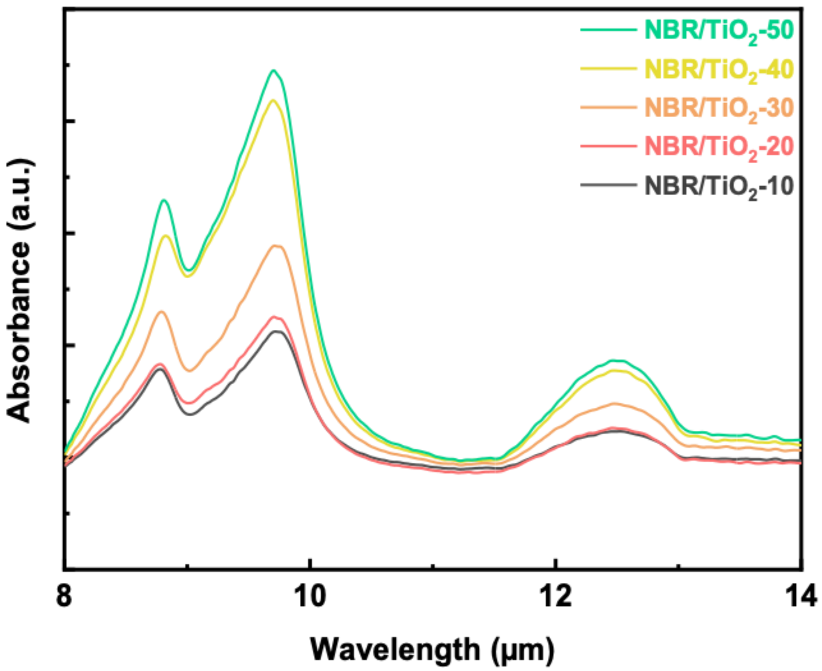

Figure S4. Infrared absorbance of the nanocomposite dielectric with different dopant conent.

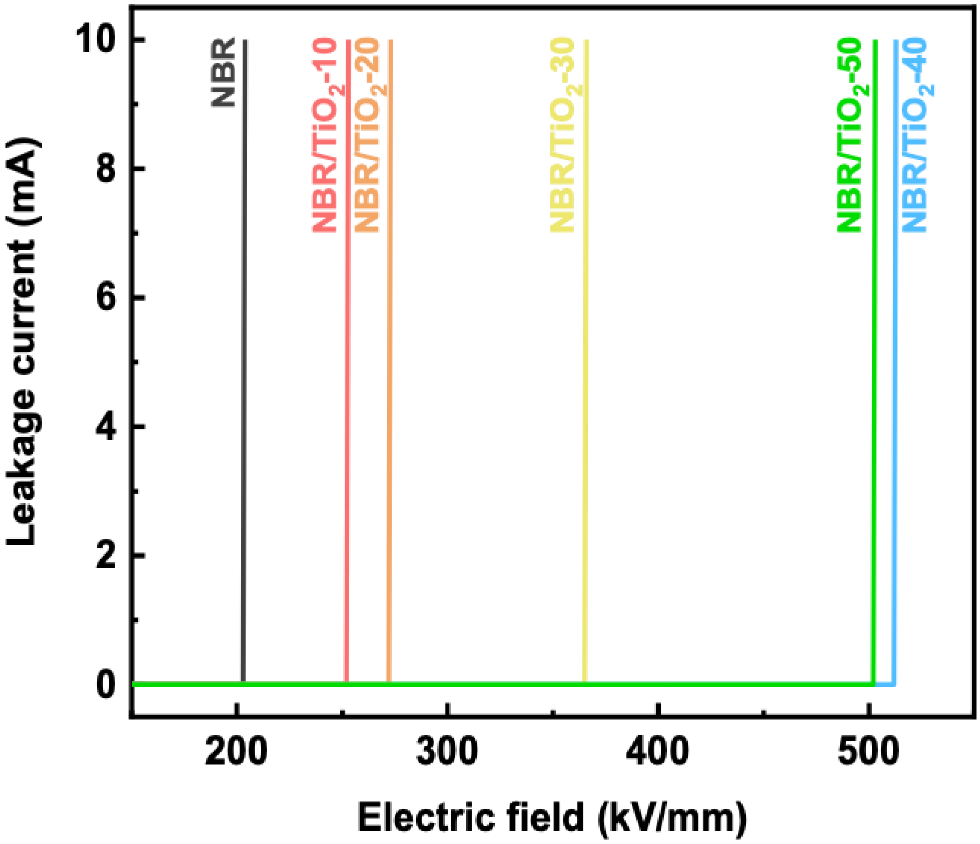

Figure S5. Comparison of the electric field of NBR and NBR/TiO_2 nanocomposite dielectrics.

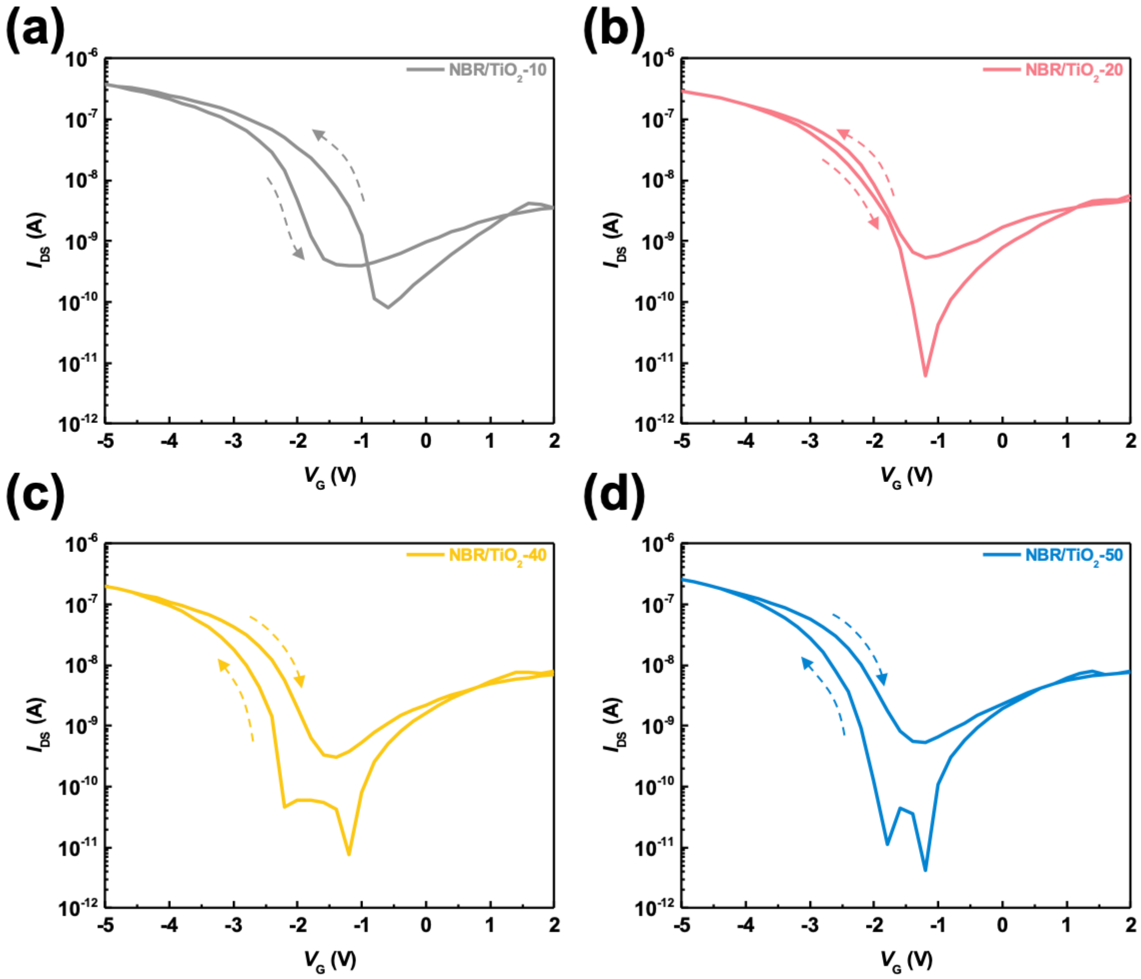

Figure S6. Effects of transfer characteristics on PDPP-TT OFETs based on the nanocomposite dielectric films of (a) NBR/TiO_2-10, (b) NBR/TiO_2-20, (c) NBR/TiO_2-40, and (d) NBR/TiO_2-50.

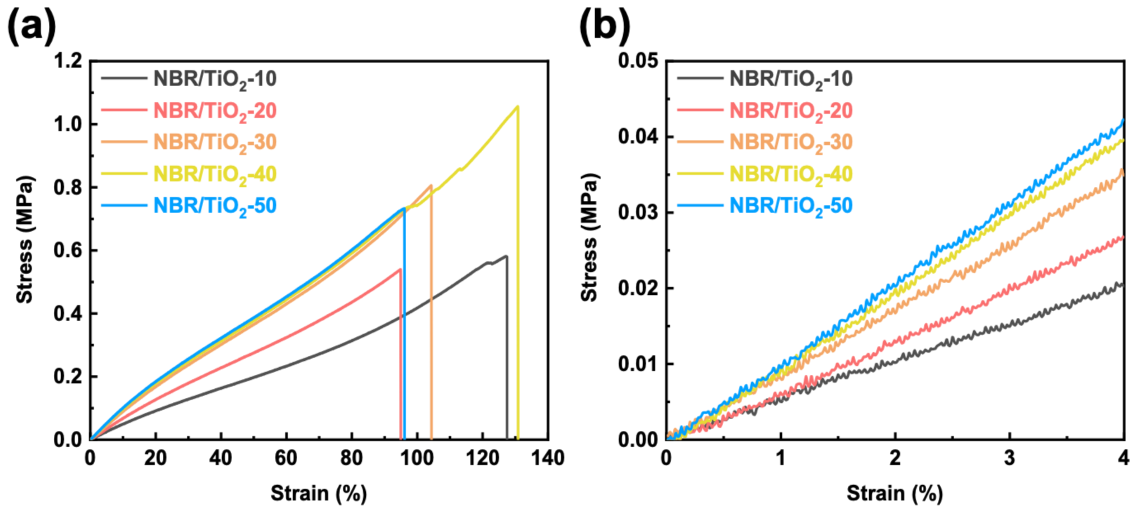

Figure S7. Stress-strain curves of nanocomposite dielectric with different TiO_2 contents. (a) Stretch to fracture point. (b) Magnified linear region before strain 4%.

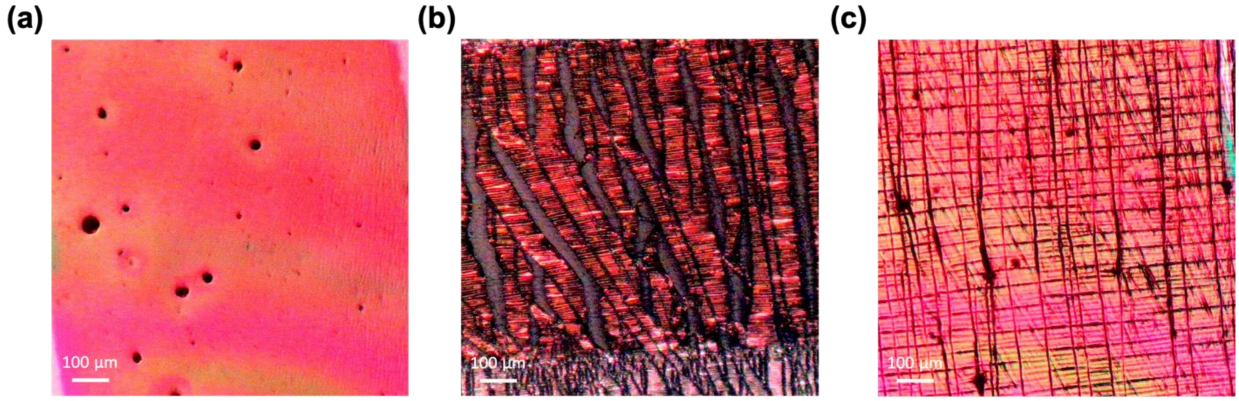

Figure S8. Optical microscopy images of the top electrode of the stretch capacitor at (a) 0% strain, (b) 100% strain, and (c) the released state.

Table S1. Summary of electrical characteristics of PDPP-TT OFETs based on nanocomposite dielectrics.

**Dopant (vol%)**	$\mu^{avg.}$ ($cm^2V^{-1}s^{-1}$)	$\mu^{Max.}$ ($cm^2V^{-1}s^{-1}$)	g_m (μS)	V_{TH} (V)	I_{on}/I_{of}
**0**	$(25.6\pm2.32)x10^{-2}$	0.289	(0.61 ± 0.05) $x10^{-1}$	-1.68±0.22	5.47 $x10^5$
**10**	$(9.83\pm0.55)x10^{-2}$	0.106	(1.27 ± 0.09) $x10^{-1}$	-0.92±0.05	4.93 $x10^3$
**20**	$(11.3\pm0.91)x10^{-2}$	0.122	(1.63 ± 0.32) $x10^{-1}$	-1.61±0.24	1.65 $x10^4$
**30**	$(13.2\pm0.65)x10^{-2}$	0.140	(2.02 ± 0.16) $x10^{-1}$	-2.10±0.13	3.24 $x10^5$
**40**	$(8.43\pm0.87)x10^{-2}$	0.289	(1.33 ± 0.11) $x10^{-1}$	-2.12±0.06	7.60 $x10^4$
**50**	$(8.47\pm0.78)x10^{-2}$	0.0929	(1.60 ± 0.15) $x10^{-1}$	-2.12±0.05	2.01 $x10^4$

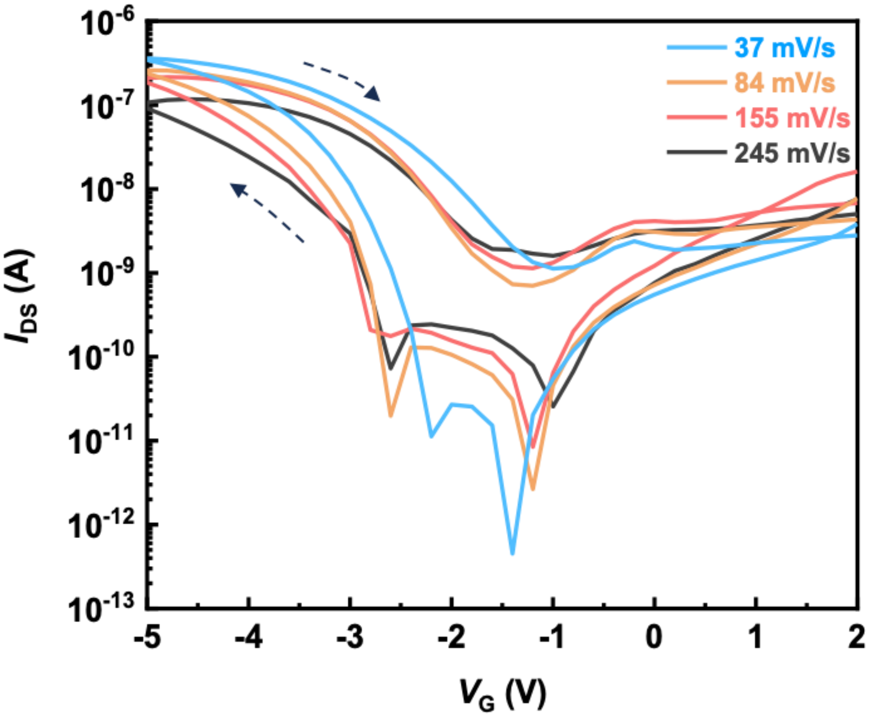

Figure S9. The influence of transfer characteristics with control scan speed on PDPP-TT OFETs based on NBR/TiO_2-30.
